# Commentary on: “Does caffeine enhance bowel recovery after elective colorectal resection? A prospective double-blinded randomized clinical trial” Tech Coloproctol. 2021 Apr 26. doi: 10.1007/s10151-021-02450-7

**DOI:** 10.1007/s10151-021-02489-6

**Published:** 2021-06-25

**Authors:** P. K. Korolkiewicz

**Affiliations:** Medical University of Gdansk, M. Skłodowskiej-Curie 3a, 80-210 Gdańsk, Poland

Dear Sir,

It is with great interest that I read the results of the study performed by Parnasa et al*.* [1], which evaluated the safety and efficacy of orally administered caffeine citrate in patients undergoing abdominal surgery.

While the paper confirmed the beneficial effect of caffeine on postoperative gastrointestinal motility, I would like to hypothesize that the results could have been optimized by a simple modification of the drug administration schedule. It is well-known fact that any pharmacological compound exerts its most potent pharmacologic effect after achieving a steady-state concentration. The 50% of the steady-state concentration is reached after one half-life (*t*_1/2_), 75% after two *t*_1/2_, and over 90% after four *t*_1/2_ [2]. The mean serum caffeine half-life for the orally ingested caffeine solution in healthy subjects equals 5.7 h [3]. Therefore, most of the patients in the study will have reached the steady state approximately 23 h after dosing initiation. Thus, the relatively modest effects observed by the authors could have been more pronounced if the caffeine “loading” was initiated prior to hospital admission and continued as described in the study protocol. This hypothesis might be worth taking into account, while further exploring the effects of caffeine in enhanced recovery after surgery protocols.

Additionally, based on the information in the paper, it is not clear whether both treatment arms were balanced with respect to the administration of nonsteroidal anti-inflammatory drugs (NSAIDs) as a part of postoperative analgesia. This confounding factor is especially important in the light of the ketorolac effect on the enhancement of normal bowel function recovery after surgical intervention. Stratification according to whether the patients have or not have taken NSAIDs as analgesics could have potentially taken care of the variable.

## Data Availability

Not applicable.

## References

[1] Parnasa SY, Marom G, Bdolah-Abram T, Gefen R, Luques L, Michael S, Mizrahi I, Abu-Gazala M, Rivkind AI, Mintz Y, Pikarsky AJ, Shussman N (2021). Does caffeine enhance bowel recovery after elective colorectal resection? A prospective double-blinded randomized clinical trial. Tech Coloproctol.

[2] Holford NHG, Katzung BG (2018). Pharmacokinetics & pharmacodynamics: rational dosing & the time course of drug action. Basic and clinical pharmacology.

[3] Statland BE, Demas TJ (1980). Serum caffeine half-lives. Healthy subjects vs. patients having alcoholic hepatic disease. Am J Clin Pathol.

